# Hydatids of the Liver

**Published:** 1828-11

**Authors:** 


### HYDATIDS OF THE LIVER.

WATERY and serous, as well as bilious, sanguineous, or purulent collections of the liver, were until lately looked upon as very obscure diseases, which frequently remain unknown until death. M. RECAMIER has cleared up the diagnosis in a very great degree. He forms so accurate a judgment of these affections, that strangers, astonished at his success, have attributed it to a species of *medical instinct,* which at first teaches him the nature of the disease. The following case will give an idea of the nature of the cysts of the liver, and the mode of operating practised by M. Recamier in order to effect their cure.

—— Marcon, thirty-eight years of age, followed a sedentary employment in one of the lowest quarters of Paris. When interrogated as to his former state of health and other circumstances, he said that he had twice suffered from tertian fever, and about five years ago had a severe attack of illness, the nature of which he was ignorant of. For about two years he had experienced an abdominal affection, that gave him great uneasiness. At that period he remarked a small tumor about the epigastric region: it disappeared, however, according to his account, and only again became visible about three weeks before his admission into the Hotel Dieu, which was on the 21st June.

The epigastrium began to swell about the end of May, but without pain: in fact, the formation of these cysts is seldom accompanied by any previous inflammation, and this is the case with regard to many other affections of the liver, and accounts for the frequent errors of diagnosis that are committed. Marcon perceived the tumefaction to augment considerably during the eight days previous to his admission into the hospital, and then lancinating pains in this region were first felt. On the 17th June he began to vomit every kind of food, generally a few minutes after swallowing it.

On the 20th June, the tumor was found to be very painful to the touch; but the patient had no fever, and, what is of importance in the diagnosis, he declared that he had not experienced any feverish attack from the commencement of the disease.—We must here remark, that the tertian which the patient had been affected with a long time before, appeared to have no connexion with the present malady.

On the 21st, the tumor had become still larger, and the pains extended to the navel. Twelve leeches were applied, and the patient was put into a warm bath. From the 23d to the 26th, the symptoms became more serious; the sleep was interrupted, and violent pains were felt throughout the whole extent of the tumor. Twelve more leeches were applied, warm baths employed, and two ounces of castor-oil given to overcome the constipation.

On the 27th, the patient was better. The tumor occupied the whole epigastrium, and it was easy to trace its boundaries inferiorly. M. Recamier declared the nature of the tumor, and, after having employed percussion several times, proposed to make a puncture.

M. Recamier calls his exploratory puncture a true acupuncture, on account of the smallness of the instrument which he employs. The fluid which issues forth shews him the precise nature of the affection; and, upon seeing the liquor that escaped in this case, he confirmed the diagnosis he had already given. A piece of potash was applied, according to the method of the Professor, in order to determine the adhesive inflammation. The day following, an oval-shaped eschar was produced, the largest diameter of which was from above downwards. Barley water was precribed for the patient's drink, and on the 29th another piece of potash was applied.

On the 1st of July, two ounces of castor-oil were required, to remove constipation, and a third application of the caustic was made. The pain in the cyst augmented, and fever came on. The eschar had not fallen out on the 7th, but the neighbouring inflammation having developed itself properly, a tolerably deep incision was made in the whole line with the bistoury. This gave vent to about half a pint of serosity slightly turbid. The day after, about a pint escaped. The patient, however, continued to suffer great pain, and complained of general uneasiness.

In all such cavities, M. Recamier considers it as highly important to prevent the introduction of air. It is the same in the synovial cavities and in the pleura, after the operation for empyema: if the air gains access, the discharge puts on a bad character, hectic fever becomes lighted up, and the patient perishes.

A sufficient quantity of Eau de Guimauve was therefore injected into the cavity, to fill it. The patient from this period found himself much better: the fever entirely ceased, and the abdomen became less tender upon pressure. Emollient drinks, baths, and cataplasms, were the only remedies employed.

On the 12th, the tumor was dispersed; and on the following day the amendment was still more evident.

On the 5th of August, the belly was still a little painful, and appeared larger than natural, and its sensibility was yet considerable in the epigastric region. A fresh puncture was made, and immediately a fluid of the most fetid odour escaped: hydatids were voided with the fluid which was contained in the cyst. The day following, the patient found himself much better, and some little time afterwards his spirits began to revive. Every day the dressing was performed with great nicety; the artificial opening was preserved by means of sponge lint, and the quantity of fluid injected became much smaller. The parietes of the cyst were discharged in fragments with the fluid, which afterwards was strongly tinged with yellow.

The state of the patient is now satisfactory: the appetite begins to revive, he lies upon the side and sleeps all night, and the capacity of the cyst diminishes every day.

